# Seed Disinfection Treatments Minimized Microbial Load and Enhanced Nutritional Properties of Fenugreek Sprouts Which Alleviated Diabetes-Negative Disorders in Diabetic Rats

**DOI:** 10.3390/nu16162635

**Published:** 2024-08-10

**Authors:** Abeer A. Dahab, Hala M. Bayomy, Hemat S. Abd El-Salam, Seham E. Almasoudi, Nawal A. Ozaybi, Gehan A. Mahmoud, Amira K. G. Atteya, Rasha S. El-Serafy

**Affiliations:** 1 Medicinal and Aromatic Plants Research Department, Horticulture Research Institute, Agricultural Research Center, Giza 12619, Egypt; hemat.sameh@yahoo.com; 2 Food Science and Nutrition Department, Faculty of Science, University of Tabuk, Tabuk 71491, Saudi Arabia; hm.mohamed@ut.edu.sa (H.M.B.); salmasoudi@ut.edu.sa (S.E.A.); n.ozaybi@ut.edu.sa (N.A.O.); 3 Fruit Crops Handling Research Department, Horticulture Research Institute, Agricultural Research Center, Giza 12619, Egypt; geh-an.ahmed.11@hotmail.com; 4 Horticulture Department, Faculty of Agriculture, Damanhour University, Damanhour 22516, Egypt; amira.khames@agr.dmu.edu.eg; 5 Horticulture Department, Faculty of Agriculture, Tanta University, Tanta 31527, Egypt

**Keywords:** disinfection, diabetes, fenugreek sprouts, microwave radiation, antioxidants

## Abstract

Sprouts are an attractive food product that contains high amounts of nutritional substances and has pro-health features. Sprout consumption has strongly increased despite its potential risk to health due to its microbial load. Both the safety and shelf life of sprouts may be negatively affected by a high microbial load. To reduce the microbial contamination in sprouts before consumption, the initial microbial load on the seeds needs to be controlled. Many herbal sprouts have been recommended for diabetes, and fenugreek is one of these sprouts. Thus, the current experiment aimed at disinfecting fenugreek seeds using microwave (5, 10, and 20 s) and hot water (30, 45, and 60 s) treatments for different durations. The best-disinfected sprouts with the highest nutritional properties were used to evaluate their influence on streptozocin-induced diabetic rats in comparison with fenugreek seed feeding. Microwave treatments showed the highest sprout length, fresh weight, total free amino acids, antioxidants, reducing sugars, and total phenols. Additionally, microwave seed treatments showed the lowest bacteria and mold counts on sprouts produced relative to hot water treatments, and the best seed treatment was a microwave for 20 s, which gave the best values in this respect. Feeding diabetic rats with different fenugreek seeds or sprout rates (0, 5, 7.5, and 10% *w*/*w*) improved body weight, restricted the growth of glucose levels, lowered total cholesterol and triglycerides, and improved HDL compared with the positive control group, and fenugreek sprouts at higher rates showed the maximum improvements in blood glucose, total cholesterol, and triglycerides. Treating fenugreek seed with microwave radiation for 20 s to disinfect the seeds before sprouting is recommended for lowering the microbial load with optimum nutritional and antioxidant activity, and feeding diabetic rats with these sprouts at the rate of 7.5 and 10% had promising effects on hyperglycemia and associated disorders.

## 1. Introduction

Fenugreek (*Trigonella foenum-graecum* L.) is an important medicinal plant, grows as an annual herb, and is a member of legume seeds. It is cultivated all over the world and originated in Greece. Fenugreek seeds have significant nutritional and therapeutic importance due to their content of nutritional components and bioactive ingredients [1], including carbohydrates (45–60%), proteins (20–30%), fixed oil (5–10%), alkaloids, steroidal saponins, phenolics, and flavonoids, in addition to volatile oils, minerals, and vitamins [2]. Therefore, it is used in the pharmaceutical industry as a steroidal diosgenin source [3], and an antioxidant, antidiabetic, anti-lithogenic, antimicrobial, anticarcinogenic, hypocholesterolemic, and immunological enhancer [4].

Fenugreek has been shown to have a hypoglycemic effect due to its content of trigonelline and fenugrecin alkaloids, as well as the effect of its soluble fibers (e.g., glucomannan) and amino acids (such as 4-hydroxyisoleucine) in stimulating the pancreas to excrete insulin [5]. It also has resulted in a decrease in blood glucose levels in rats that were not insulin dependent as well as in healthy individuals [6]. The addition of seed powder to the diet of diabetic rats induced with alloxan has a positive impact on the levels of glycolytic, gluconeogenic, and lipogenic enzymes, ultimately leading to the restoration of glucose homeostasis [7]. Despite the fact that bioactive substances are found in small quantities in foods, they are vital nutritional components whose consumption promotes numerous health benefits and guards against chronic degenerative diseases [8]. Annually, there is an increase of 10% in bioactive component consumption and their related food products [9]. Several clinical reports state that consuming foods high in antioxidants is associated with a lower risk of degenerative disease occurrence.

Sprouting is a high-impact procedure that increases the amount of available nutrients while lowering the concentration of anti-nutrients at a low cost, with possible health advantages [10]. Sprouts are the byproduct of seeds that are developed in water and collected before true leaves appear. These sprouts are consumed whole, including the seeds [11]. Although consuming different seed sprouts has increased and spread globally due to their high nutritional content and accessibility [11], many consumers are exposed to illness due to the contamination of sprouts. In 1995, the Food and Drug Administration (FDA) in the United States considered sprouts to be a source of foodborne illness and recognized sprouts as a Potentially Hazardous Food [11]. Sprouts can serve as a good food source for bacteria that cause deterioration and pathogenicity. Three distinct stages comprise the germination process: disinfection, steeping, and sprouting. Before seed germination, disinfection is conducted to lower the microbial load of the seeds. Formaldehyde, ethanol, or sodium hypochlorite are the common subjects used for seed disinfection [12]. However, the disinfection phase is skipped because of the possible negative effects and hazards to the food safety of the seeds. The seed sprouting occurred in controlled environments with moist, warm, and nutrient-rich conditions, and this environment is ideal for microbial activity and growth. If the seeds have a microbial load or are not disinfected, the high microbial load can lower the sprouts’ shelf life and raise the infection probability [13].

Diabetes is a metabolic defect identified by hyperglycemia due to irregularities in the secretion and action of insulin [14]. Because of the decreased antioxidant levels and increased formation of reactive oxygen species, persistent hyperglycemia causes microvascular problems (such as neuropathy and nephropathy) as well as macrovascular problems (mostly cardiovascular problems) [15]. The worldwide incidence of diabetes mellitus is rising and is predicted to reach 592 million people by 2035, mainly as a result of increasing rates of obesity [16]. Although the etiology, clinical presentation, and disease prevalence of type 1 and type 2 diabetes differ, both kinds of diabetes include secondary consequences such as endothelial dysfunction. Type 2 diabetes is more common in older adults than children, which occurs due to a problem in the way the body uses and regulates glucose as a fuel. Diabetes type 2 has two major issues: (1) the pancreas does not create enough insulin (the hormone that controls the flow of sugar into cells), and (2) cells respond poorly to insulin and consume less sugar. The majority of diabetes problems are incurable, meaning that the health system and society bear a heavy financial cost.

Throughout history, herbal medications have been widely used to cure a variety of illnesses [17,18,19,20,21,22]. Researchers are attempting to assess both the potential positive and negative effects of these natural compounds on human health because it is still unclear how exactly these products are characterized and how they work [23,24,25,26]. Patients prefer to use botanicals over various medications, including insulin analogues, thiazolidine, biguanides, sulphonylureas, dipeptidyl peptidase-4 inhibitors, and α-glucosidase inhibitors, because of the side effects and higher cost of these treatments [15]. Several medicinal plants, including fenugreek, are used as diabetes treatments due to their ability to decrease glucose and lipid levels by inducing insulin secretion, inhibiting glucosidase activity, elevating GLUT4 expression [27], and inhibiting gluconeogenesis [28], in addition to activating the AMP-activated protein pathway [29].

Therefore, to minimize the microbial load of sprouts before consumption, the initial microbial load of the seeds should be controlled [30]. Overlapping this problem can be performed by many strategies, including electromagnetic field applications. Microwave radiation application in food processing to inactivate bacterial growth and different microorganisms is an important technique [31]. Electromagnetic fields induce physiological alteration in plant cell structure, increase nutritional value, and boost crop yields [32]. Physical conditions including light, temperature, disinfection, genotype [33], and seed chemical composition caused a variation in the germination and antioxidant features of seeds [34], including fenugreek. Fenugreek seed sprouts showed a better nutritional profile and a lower fiber level, making them easier to absorb and digest [35], and a higher antioxidant level with more antidiabetic efficiency than their boiled seeds [36]. The release or increased bioavailability of bound antioxidants during sprouting could be the cause of these effects.

Therefore, the objective of this study is to evaluate the most common procedure for fenugreek seed disinfection using microwave and hot water at different durations to minimize the microbial load on the sprouts prior to consumption with optimal nutritional value, and then using these sprouts in the diet of streptozocin-induced diabetic rats to evaluate their effect on serum glucose, total cholesterol, triglycerides, HDL, and kidney markers.

## 2. Materials and Methods

### 2.1. Sprout Experiment

#### 2.1.1. Seed Treatments

Fenugreek (*Trigonella foenum-graecum* L.) seeds were provided from the Horticulture Research Institute, Agricultural Research Center, Giza, Egypt. The seeds were washed three times with distilled water, dried, and divided into seven groups. Each group contained 2 g of seeds, which were put in 9 cm glass culture dishes. Three groups of seeds were exposed to irradiation using a microwave (230 volts, 50 Hz, 900–1300 W) for 5 s (M5s), 10 s (M10s), and 20 s (M20s); the other three groups were exposed to hot water (80 ± 1 °C) for 30 s (W30s), 45 s (W45s), and 60 s (W60s), while the last group was not treated, as a control group. After treatment, the seeds for each group were separately soaked in sterilized water for 24 h. During the soaking period, the water was changed one time. After pouring off the soaking water, the seeds were incubated for 4 days at 85% RH and 24 ± 2 °C in a seed germination incubator. Throughout the incubation period, the seeds were rinsed with 8 mL of sterile distilled water every 12 h. The sprouts were promptly removed after germination and stored at −80 °C until use and biochemical analysis.

#### 2.1.2. Microbial Load Determination

Three replicates were performed to assess the number of bacteria and fungi on the fenugreek seeds and on the sprouts produced from the treated seeds. In total, 1 g of samples was mixed with 9 mL of sterile distilled water and homogenized for 1 h using a horizontal shaker (MK201D, Yamato Scientific Co., Ltd., Shanghai, China). After that, 10–4 serial dilutions were made. For uniform distribution, 1 mL of a 10–4 dilution was pipetted into sterile Petri plates along with potato dextrose agar for fungus at 45 °C and molten nutrition agar for total bacteria. Then, Petri plates with molten nutrition agar were incubated for 24 h at 37 °C, and the colonies of bacteria were enumerated. The Petri plates along with potato dextrose agar media were incubated for 5 days at 25 °C, and the colonies of fungus were enumerated [37]. The average number of colonies that developed on the plates was noted. The colony-forming units (log cfu g^–1^ fresh weight) were used to represent the total microbial count.

#### 2.1.3. Biochemical Analysis of Fenugreek

Total free amino acids in seeds and sprouts were estimated according to the procedure outlined by Yemm and Cocking [38] (1955) using the ninhydrin reagent technique. Reducing sugar in seeds and sprouts was extracted as mentioned by Kawamura [39] and determined by the 3,5-dinitrosalicylic acid (DNSA) method, according to Krivorotova and Sereikaite [40]. Antioxidant activity (% as DPPH) was determined based on the radical scavenging ability of reacting with a stable DPPH-free radical, according to Chen et al. [41]. Total phenols were evaluated in dried fenugreek seeds or sprouts, extracted using 80% ethanol and determined according to the colorimetric method described by Boateng et al. [42].

### 2.2. Animal Experiment 

This experiment was performed as given by the standards of animal care and approved by the Ethics Committee of Tanta University, Egypt, during the period of March–April 2022. In total, 48 male Wistar albino rats were acquired from the Faculty of Veterinary Medicine, Cairo University; their weights ranged from 165 to 180 g. The rats were put in cages (22 °C and 45–65% HD) for one week before the beginning of the experiment and were provided with unlimited ordinary rat pellet diets and water.

#### 2.2.1. Diabetes Model

Before beginning streptozocin (STZ) therapy, all rats were weighed, received water as normal, and fasted for 12 h. Then, the rats were injected intraperitoneally with STZ, which was prepared by mixing it with 0.5 mL sodium citrate buffer (pH 4.5) at a rate of 35 mg/kg body weight [43] for all rats except the negative control group, which was injected intraperitoneally with 0.5 mL of citrate buffer (pH 4.5) [44]. After that, the rats were fed and kept on 10% (*w*/*v*) sucrose water for one day, and then tap water was used [44]. One week after STZ injection, fasting glucose levels were estimated for each rat, and rats above 200 mg/dL were considered in the study [45].

#### 2.2.2. Fenugreek Treatments

The rats were divided into eight groups randomly (n = 6): group 1: served as a nondiabetic and untreated control group (Negative control), group 2: functioned as a diabetic control group (Positive control), group 3: diabetic rats receiving 5% (*w*/*w*) fenugreek seeds powder (Diabetic FS1), group 4: diabetic rats receiving 7.5% (*w*/*w*) fenugreek seeds powder (Diabetic FS2), group 5: diabetic rats receiving 10% (*w*/*w*) fenugreek seeds powder (Diabetic FS3), group 6: diabetic rats receiving 5% (*w*/*w*) fenugreek sprouts of microwave treatment of M10s (Diabetic FSP1), group 7: diabetic rats receiving 7.5% (*w*/*w*) fenugreek sprouts of microwave treatment of M10s (Diabetic FSP2), and group 8: diabetic rats receiving 10% (*w*/*w*) fenugreek sprouts of microwave treatment of M10s (Diabetic FSP3). Fenugreek seeds were crushed using an electric grinder, and the powder was added to the standard diet, which was bought from the forage market. The standard diet contains starch (67.0%), casein (15.0%), salts (4.0%), wood fiber (5.0%), corn oil (8.0%), and vitamins (1.0%), and then a little distilled water was added [7], formed into pellets, dried, and kept in plastic containers at 5 °C. The fresh sprouts were crushed and mixed with the standard diet in the same manner as fenugreek seeds.

#### 2.2.3. Blood and Kidney Markers Analysis

After 30 days of the feeding period, the rats’ body weight was measured, and blood and kidney marker samples were collected for biochemical analysis. Glucose was analyzed according to Sasaki [46] method using O-toluidine, total cholesterol was estimated according to Richmond [47], triglycerides were assessed using the Trinder [48] method (mgdL^-1^), and high-density lipoprotein (HDL) cholesterol (mgdL^-1^) was measured using the Richmond [47] procedure. Kidney markers of urea, uric acid, and creatinine were estimated by alkaline picrate method.

### 2.3. Statistical Analysis

Data pooled were statistically analyzed using the COSTAT version 6.4. The Tukey test with a 0.05 probability was used to determine whether there was a significant difference between the mean values [49]. The results were expressed as mean ± SE, n = 6. 

## 3. Results

### 3.1. Germination and Sprouts Physical Traits

**Figure 1:**
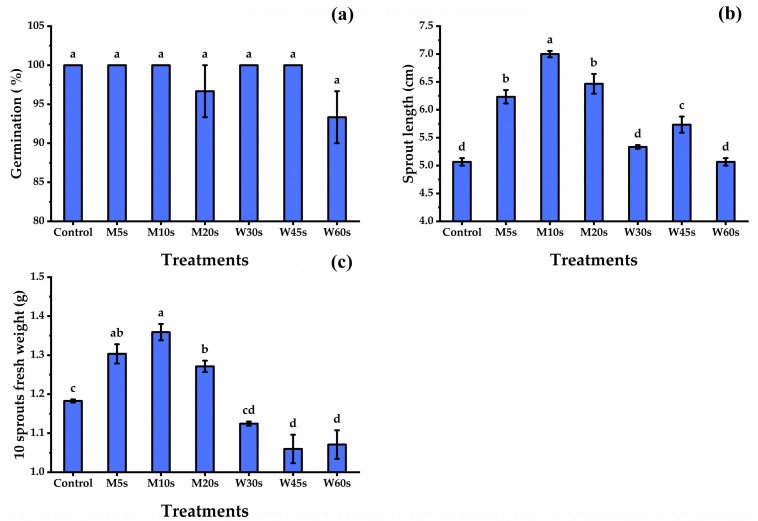
Germination% (**a**), sprout length (**b**), and 10 sprouts fresh weight (**c**) of fenugreek sprouts in response to microwave and hot water seed disinfection treatments. M5s, microwave for 5 s; M10s, microwave for 10 s; M20s, microwave for 20 s; W30s, hot water for 30 s; W45s, hot water for 45 s; and W60s, hot water for 60 s. All data denote mean ± S.E. Means with different letters significantly differed, using Tukey test at *p* ≤ 0.05.

The germinated seeds of all disinfection treatments showed nonsignificant differences regarding germination percentage, as all seeds appeared to have full germination expect for the treatment of W60s, which showed a decrease of about 6.7% compared with other disinfection treatments (Figure 1). Concerning the sprouts’ features, seed treatments caused many changes in the sprouts’ physical traits as the sprouts’ length became taller as affected by hot water and microwave treatments relative to the control seeds, and the highest length was exhibited by the seeds treated with M10s, which was 24.6% taller than the control seeds, followed by M20s and M5s, while the lowest length was given by the control seeds. Hot water treatments showed a significant and gradual increase in sprout length until the W45s treatment, which decreased after that. The fenugreek seeds exposed to microwave treatments showed an increase in the sprouts’ fresh weight compared with hot water and control treatments, and the highest fresh weight of 10 sprouts was produced by the M10s treatment, followed by the M5s, but the lowest fresh weight values were given by the hot water treatments, which reached the lowest values by the W45s treatment.

### 3.2. Biochemical Analysis

**Figure 2:**
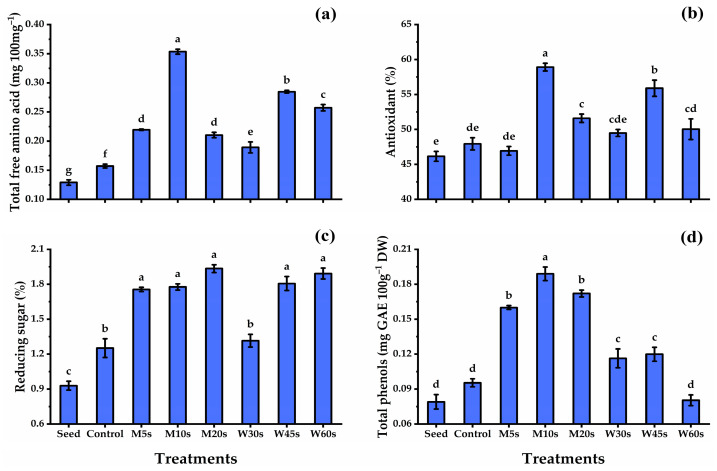
Total free amino acid (**a**), antioxidant (**b**), reducing sugars (**c**), and total phenols (**d**) of fenugreek sprouts in response to microwave and hot water seed disinfection treatments. M5s, microwave for 5 s; M10s, microwave for 10 s; M20s, microwave for 20 s; W30s, hot water for 30 s; W45s, hot water for 45 s; and W60s, hot water for 60 s. All data denote mean ± S.E. Means with different letters significantly differed, using Tukey test at *p* ≤ 0.05.

The data presented in Figure 2 showed the biochemical analysis of fenugreek seeds and sprouts as affected by different disinfection treatments. The fenugreek seed content of total free amino acids and reducing sugar significantly exhibited lower values compared with different fenugreek sprouts. Different disinfection treatments exhibited great improvements in the biochemical analysis of the sprouts produced. Fenugreek seeds subjected to M10s treatment significantly gave the maximum total free amino acid value (0.354 mg 100 mg^−1^), while the lowest value in this regard was exhibited by the untreated fenugreek seeds (0.129 mg 100 mg^−1^). Hot water treatments showed an enhancement in total free amino acid values compared with the untreated seeds and control sprouts, where the W45s sprouts presented the highest value in this regard. The presented results in Figure 2b,d show that the M10s treatment recorded the highest antioxidants (58.92%) and total phenols (0.189 mg GAE 100 mg^−1^ DW) values, followed by fenugreek sprouts at W45s for antioxidants and M20s for total phenols. On the other hand, the lowest antioxidants and total phenol content were exhibited by untreated seeds, which means that seed treatments were an effective tool in increasing the antioxidant activity of the sprouts produced. All disinfection treatments led to an enhancement in the reducing sugar content, and increasing the sprout duration from 5 s to 20 s for microwave and from 30 s to 60 s for hot water treatments led to an increase in the reducing sugar content. In this regard, the highest reducing sugar value was given by the M20s treatment, while the lowest reducing sugar content was given by untreated fenugreek seeds.

### 3.3. Microbial Load

**Figure 3:**
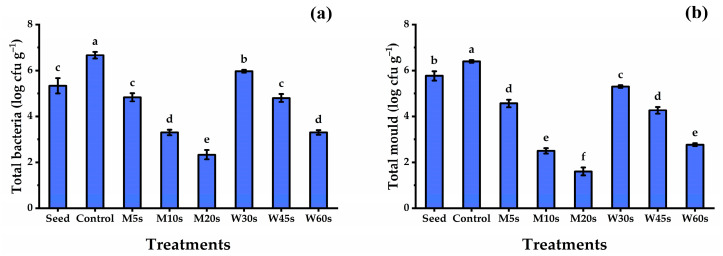
Total bacteria (**a**) and total mold (**b**) of fenugreek sprouts in response to microwave and hot water seed disinfection treatments. M5s, microwave for 5 s; M10, microwave for 10 s; M20s, microwave for 20 s; W30s, hot water for 30 s; W45s, hot water for 45 s; and W60s, hot water for 60 s. All data denote mean ± S.E. Means with different letters significantly differed, using Tukey test at *p* ≤ 0.05.

The initial total microbial count estimated from the untreated fenugreek seeds was 5.4 log CFU g^−1^ FW for total bacteria and 5.8 log CFU g^−1^ FW for total mold (Figure 3). On the other hand, most of the disinfection applications were effective in substantially decreasing the microbial loads on the fenugreek sprouts. As indicated in Figure 3, the lowest total bacterial count (2.33 log CFU g^−1^ FW) and total mold (1.60 log CFU g^−1^ FW) across all the disinfection treatments were given by M20s-treated seeds, followed by M10s and W60s treatments, with nonsignificant differences among them. The highest microbial load was observed by control sprouts, which gave 6.67 log CFU g^−1^ FW for total bacteria and 6.40 log CFU g^−1^ FW for total mold. These results suggest that the microwave treatment at M20s was the most effective seed disinfection approach compared to other treatments.

### 3.4. Rats Body Weight

The body weight performance of negative and positive diabetic rats tabulated in Table 1 revealed that the negative diabetic rats significantly showed the highest body weight gain (37.7 g), while positive diabetic rats exhibited negative body weight changes as their weights decreased by 5.3 g compared with their initial weights. On the other hand, all other experimental groups exhibited an improvement in their body weight performance to different degrees relative to positive diabetic rats. The diabetic rats treated with FS1 exhibited the lowest gain in their body weight, but the diabetic rats treated with FSP2 and FSP3 exhibited the highest improvement in their body weight, but with nonsignificant differences among them.

### 3.5. Blood and Kidney Markers Analysis

The data presented in Table 2 show that the fasting blood glucose levels of different diabetic rats were elevated significantly compared with the negative control rats. The highest elevation in fasting blood glucose level (325.7 mg/dL) was recorded by the positive control rats, but this value was decreased following different fenugreek feeding treatments. The highest reduction in the fasting blood glucose level (117.1 mg/dL) was recorded by the diabetic rats who received fenugreek sprouts at 7.5% in their diet (Diabetic FSP2), followed by diabetic FSP3 rats and FSP1 rats, with no significant difference among them. The results in the same table point out the changes in the lipid profile. Total cholesterol and triglyceride levels of the diabetic rats increased significantly, whereas high-density lipoprotein (HDL) levels decreased significantly in comparison to the negative control rats. Each of the fenugreek seeds or sprouts lowered the lipid profile, but, to some extent, fenugreek sprouts had the best effect on lowering the lipid profile.

The administration of the fenugreek sprouts significantly decreased serum total cholesterol and triacylglycerol in diabetic rats compared with nondiabetic control rats, and the lowest values in this respect were recorded by FSP2-rats, who gave 94.1 and 56.4 mg dL^−1^ for total cholesterol and triacylglycerol, respectively. Regarding the nondiabetic control rats group, they had the lowest total cholesterol and triacylglycerol values, but these values were maximized by the positive rats group. It is clearly shown that a significant decrease in HDL values was shown by the diabetic rats and all fenugreek treatments, but the oral administration of FSP2 restricted the reduction of HDL values. The results presented in Table 3 show that uric acid, urea, and creatinine values increased in diabetic control rats when compared with nondiabetic control rats. The fenugreek seeds and sprouts treatments significantly lowered urea, uric acid, and creatinine values compared with the control diabetic rats. The rats group supplemented with fenugreek sprouts of FSP2 significantly recorded the lowest values in this respect relative to the other treatments.

## 4. Discussion

### 4.1. Effect of Disinfecting Treatments on Germination and Seedling Traits

Seed disinfection has been proposed as an important step in a multi-part technique to decrease the risk of illness caused by polluted sprouts. In this study, all seed treatments showed nonsignificant differences in the germination percentage. The lowest sprout length was recorded by control seeds followed by hot water treatments, but the lowest sprout fresh weight was recorded by hot water treatments. Contrary to our findings, Hermansen et al. [50] found that hot water treatments at 44, 49, and 54 °C on carrot seeds enhanced the germination rate and seedling features. The better crop was obtained by treating okra seeds in hot water for 30 min at 52 °C [51].

### 4.2. Effect of Disinfecting Treatments on Sprouts’ Biochemical Analysis

Microwave treatments showed improvements in the biochemical analysis of total phenols, antioxidants, and total free amino acids; reducing sugar content in the sprouts produced; and increasing the exposure time increased these values. Similar findings were reported by Ragha et al. [52] and Gaurilcikiene et al. [53], who indicated that extending the microwave exposure time increased the antioxidant activities in wheat seedlings. Chen et al. [54] illustrated that lower power and microwave duration decreased antioxidant activities and seedling vigor in *Isatis indigotica* fort. The biologically active ingredient yield of buckwheat has been elevated following microwave treatments [55]. The germination rate and seedling vigor of lentil seeds subjected to microwave radiation (450–730 W) for 30 s were enhanced, but these values decreased with longer exposure times of 60 and 90 s [56]. In this study, seed microwave treatments elicited the bioactive components of fenugreek sprouts. Microwave seed treatments stimulated phenolic production [34]. The nonthermal influences on seeds are induced by the close contact of the microwaves with seed tissue components or molecules as particles try to organize themselves within an electric field and reduce the potential energy [57]. As recorded by Hamada [58], wheat seedlings showed an elevation in amino acid and protein contents as affected by microwave seed treatment. Radish seeds treated with microwave radiation showed an induction in the bioactive component concentration, including a variety of enzymes involved in seed germination, improving the germination rate in addition to boosting the production of specific biological components in the seeds [57]. In plant seedlings, microwave pretreatment increases the expression of genes encoding superoxide dismutase and peroxidase isozymes, which leads to a significant increase in the seeds’ germination potential, stem length, and total mass [56,57], in addition to cell membrane integrity, which improves plant resistance to environmental stress [59]. Microwaves can enhance the quality of germinated grains, their nutritional value, and the secondary metabolite content of grains and sprouts [60]. The advantageous effects of microwave frequency on other metabolic processes, including water absorption [61], the degradation of larger phenolic and flavonoid compounds into smaller ones, and phenolic compounds released from glycosidics by irradiation caused an increase in the total phenol content [62] in fenugreek. These findings would also explain the improvement in the phenolic content due to the changes in tissue structure caused by irradiation.

### 4.3. Effect of Disinfecting Treatments on the Microbial Load

Seed treatments with hot water or microwave irradiation decreased the microbial load in fenugreek sprouts. The optimal temperature and length of seed treatment must be determined for each crop and the associated pathogen. This treatment aims to eliminate infections as far as possible without affecting seed germination. As reported by Singh et al. [63], variations in the germination rate of seeds might occur even with a 5 min variation in treatment duration. The ideal combination of temperature and time for a particular plant seed is determined by many variables that interact with the host’s heat tolerance, including the state of the outer layers, dormancy, moisture content, age, and vigor [64]. The seeds are more resistant to high temperatures the lower their initial water content is upon heating [63]. Hot water is used to destroy structural components and interfere with vital life processes, such as protein denaturation, which results in the death or inactivation of viruses, bacteria, protozoa, and other pathogens [65]. Hot water treatment for sweet pepper seeds was found to be beneficial in reducing seed-borne infections. Fungal infections were shown to be less common when bell peppers were treated with hot water at 45 °C for 15 min or 53 °C for 4 min before being stored at 8 °C [66]. Hot water treatments for bell pepper seeds led to a significant decline in seed viability, whereas there was no discernible impact on seed vigor [67]. Soaking carrot seeds in 52 °C of hot water for 25 min was effective in eliminating *Xanthomonas hortorum* pv. Carotae bacteria [68].

Microwave radiation heats biological systems dielectrically; this heat is produced when the microwave energy is absorbed by a dielectric substance (usually water) and is then converted into heat by the rotation’s internal resistance [69]. The movement of dipolar molecules causes the dissipation of energy received from microwaves due to friction [70]. The thermal theory of microwave radiation states that there is no basis for direct electromagnetic interaction with living systems that is not dependent on these temperature-mediated effects. The bioeffects of microwave radiation are only explained by differences in temperatures or temperature profiles between microwave and conventionally heated systems [70]. The duration of microwave exposure is a significant determinant of the impact of microwaves on living cells, as shown in the current as increasing the exposure period from 5 s to 20 s led to a significant reduction in the microbial count on the fenugreek sprouts. The effects of microwave irradiation are based on the effects of temperature and electromagnetism on organisms. The extent to which microwaves impact microorganism growth is mostly determined by the radiation intensity and total energy assimilated by the organisms. Thus, microwaves are most likely to have a dominant thermal effect and destroy yeast or bacteria when they are applied at high frequencies, with high energy, and over an extended period of time. Generally, seeds have a 3–6 log 10 CFU g/L microbial load [71]. Irradiation can reduce microbial contamination by about 3.18 log CFU/g [72]. According to Danilchuk and Alkhateeb [73], the lethal impact of microwave radiation was significantly reduced in arid environments and manifested itself only after an extended duration of exposure.

### 4.4. Effect of Feeding Treatments on the Body Weight and Markers

Fenugreek is frequently consumed throughout the world. Here, we present a comprehensive examination of the effects of fenugreek seed or sprout feeding on body weight, blood, and kidney markers. Diabetic rats receiving fenugreek seed or sprout exhibited a gain in their body weight, while positive control rats presented a progressive decrease in their weight (Table 1). The gradual rise in body weight following fenugreek treatments indicates that fenugreek, especially when taken in high doses, may be able to reduce the toxicity of STZ [74]. Numerous biochemical changes occur in the seeds during sprouting, such as an increase in the bioavailability of macro- and micro-elements [75]. The highest weight gain was observed with the highest dose of sprout treatment, and this may be explained by the fact that sprout treatments led to better utilization of nutrients in the diet and thus a weight gain [76].

In this study, fenugreek treatments elevated HDL concentration while reducing triglycerides and total cholesterol. The reduction in triglycerides and total cholesterol following fenugreek treatments may be due to the depression in non-esterified fatty acids in diabetic rats, as these acids have an impact on vascular alterations and platelet aggregation through improving the prostacyclin rate in plasma [76]. Fenugreek is rich in bioactive fiber, which has an important role in retarding fat and carbohydrate absorption that participates in the hypolipidemic effect [77]. Additionally, fenugreek has trigonelline, alkaloids, and the saponin compound diasgenin, which has a role in inhibiting intestinal glucose uptake [78]. Also, the amino acids tryptophan and arginine present in fenugreek seeds have hypoglycemic and antidiabetic impacts [5]. Our results showed that treated fenugreek seeds with microwave gave higher antioxidant levels in their sprouts than other treatments, and M10s treatment gave approximately 2.7% higher antioxidant content than fenugreek seeds and control. Similar results were reported by Chand et al. [79], who revealed that fenugreek sprouts have a higher antioxidant content than fenugreek seeds. Khoja et al. [80] revealed that apigenin, one of the isoflavones in fenugreek, is more abundant in the methanolic extract of fenugreek sprouts than seeds. Apigenin has antioxidant activities and can limit or decline oxidative stress by scavenging free radicals and enhancing cellular antioxidant defense activities [81]. A decline in the triglycerides and total cholesterol accumulation in the liver and blood serum of hyperlipidemia rats following apigenin treatment was observed by Muhammed et al. [82]. Apigenin enhances hepatic LDL-C absorption and boosts the transformation of hepatic cholesterol into bile acid [83].

The pathophysiology of diabetic nephropathy is mainly influenced by oxidative stress [84]. The imbalance between the antioxidant defense system and free radicals results in an increase in free radicals [85]. Persistent hyperglycemia exacerbates oxidative stress by boosting the generation of reactive oxygen species (ROS) [86]. Excessive free radicals can cause malfunctioning kidney tissue by oxidizing various cell macromolecules once they have overcome the body’s natural antioxidant defense mechanism [87]. The kidneys subjected to hyperglycemia had lower levels of antioxidant indicators, which were restored by apigenin administration [88]. Also, Lin and Yin [89] found a decrease in the endogenous antioxidant levels in diabetic kidney cells. Hyperglycemia can boost renal function biomarkers (uric, creatinine, and urea), which are linked to glomerular atrophic changes, interstitial atrophy, and epithelial necrosis, causing diabetic nephropathy [90]. Apigenin normalizes creatinine, uric, and urea levels significantly, so this antidiabetic compound can maintain renal function in diabetic rats [88].

## 5. Conclusions

The high microbial load on seeds can decrease the safety and shelf-life of sprouts, so this microbial contamination should be minimized before the consumption of sprouts. In this regard, fenugreek seeds were exposed to different disinfection treatments using microwave and hot water, and the results obtained revealed that microwave treatment for 20 s was the best treatment in improving the sprout antioxidants with 2.7% compared with untreated seeds with minimizing the microbial load of their sprouts. Feeding streptozocin-diabetic rats with these sprouts at 7.5 and 10% enhanced their body weight, restricted the growth of glucose levels, lowered total cholesterol, triglycerides, and improved HDL, as well as enhanced kidney markers.

## Figures and Tables

**Table 1:** Effect of diabetic rats feeding with fenugreek seed powder and sprouts on the initial weight, final body weight, and changes in body weight of diabetic rats.

Treatments	Initial Body Weight (g)	Final Body Weight (g)	Changes in Body Weight (g)
Negative control	176.0 ± 1.00 ^a^	213.67 ± 0.67 ^a^	37.7 ± 0.33 ^a^
Positive control	173.0 ± 1.15 ^a^	167.67 ± 1.45 ^g^	−5.3 ± 1.20 ^f^
Diabetic FS1	176.7 ± 0.67 ^a^	182.33 ± 2.03 ^f^	5.7 ± 1.45 ^e^
Diabetic FS2	174.6 ± 0.88 ^a^	189.33 ± 1.66 ^e^	14.7 ± 0.88 ^d^
Diabetic FS3	176.0 ± 1.15 ^a^	192.33 ± 0.58 ^e^	16.0 ± 1.00 ^d^
Diabetic FSP1	175.7 ± 1.76 ^a^	196.00 ± 1.00 ^d^	20.3 ± 1.86 ^c^
Diabetic FSP2	173.3 ± 0.88 ^a^	200.33 ± 1.33 ^c^	27.0 ± 0.57 ^b^
Diabetic FSP3	176.3 ± 1.20 ^a^	205.00 ± 1.15 ^b^	28.7 ± 0.33 ^b^

Negative control: nondiabetic and untreated rats, Positive control: diabetic rats, Diabetic FS1: diabetic rats received fenugreek seed powder at 5%, Diabetic FS2: diabetic rats received fenugreek seed powder at 7.5%, Diabetic FS3: diabetic rats received fenugreek seed powder at 10%, Diabetic FSP1: diabetic rats receiving 5% fenugreek sprouts, Diabetic FSP2: diabetic rats receiving 7.5% fenugreek sprouts, and Diabetic FSP3: diabetic rats receiving 10% fenugreek sprouts. Data are presented as mean ± SE (n = 6). Values with different letters in the same column significantly differ at *p* ≤ 0.05.

**Table 2:** Effect of diabetic rats feeding with fenugreek seed powder and sprouts on the serum glucose, total cholesterol, triglycerides, and HDL of diabetic rats.

Treatments	Glucose	Total Cholesterol	Triglycerides	HDL
Unit		mg dL^−1^		
Negative control	94.9 ± 0.17 ^f^	93.57 ± 0.42 ^f^	55.96 ± 1.13 ^c^	60.87 ± 0.64 ^a^
Positive control	325.7 ± 2.1 ^a^	114.86 ± 1.06 ^a^	69.97 ± 0.85 ^a^	40.08 ± 0.41 ^f^
Diabetic FS1	278.7 ± 3.8 ^b^	112.17 ± 0.89 ^b^	66.89 ± 0.81 ^ab^	49.16 ± 0.36 ^e^
Diabetic FS2	227.9 ± 1.6 ^c^	107.23 ± 0.93 ^c^	65.26 ± 2.24 ^b^	50.93 ± 0.25 ^d^
Diabetic FS3	169.6 ± 4.2 ^d^	104.79 ± 0.50 ^d^	66.64 ± 0.51 ^ab^	52.03 ± 0.73 ^cd^
Diabetic FSP1	125.15 ± 0.6 ^e^	99.26 ± 0.39 ^e^	58.10 ± 0.13 ^c^	53.62 ± 0.34 ^bc^
Diabetic FSP2	117.1 ± 0.82 ^e^	94.08 ± 0.68 ^e^	56.36 ± 0.40 ^c^	54.74 ± 0.54 ^b^
Diabetic FSP3	121.6 ± 0.84 ^e^	97.43 ± 0.52 ^f^	56.89 ± 0.28 ^c^	53.09 ± 0.09 ^bc^

Negative control: nondiabetic and untreated rats, Positive control: diabetic rats, Diabetic FS1: diabetic rats received fenugreek seeds powder at 5%, Diabetic FS2: diabetic rats received fenugreek seeds powder at 7.5%, Diabetic FS3: diabetic rats received fenugreek seeds powder at 10%, Diabetic FSP1: diabetic rats receiving 5% fenugreek sprouts, Diabetic FSP2: diabetic rats receiving 7.5% fenugreek sprouts, and Diabetic FSP3: diabetic rats receiving 10% fenugreek sprouts. Data are presented as mean ± SE (n = 6). Values with different letters in the same column significantly differ at *p* ≤ 0.05.

**Table 3:** Effect of diabetic rats feeding with fenugreek seed powder and sprouts on uric, urea, and creatinine.

Treatments	Uric	Urea	Creatinine
Negative control	2.31 ± 0.08b ^c^	34.06 ± 0.36 ^f^	0.24 ± 0.008 ^e^
Positive control	2.86 ± 0.07 ^a^	57.87 ± 0.98 ^a^	0.38 ± 0.014 ^a^
Diabetic FS1	2.59 ± 0.12 ^b^	57.25 ± 0.29 ^a^	0.38 ± 0.003 ^a^
Diabetic FS2	2.47 ± 0.55 ^bc^	52.52 ± 0.80 ^b^	0.34 ± 0.01 ^b^
Diabetic FS3	2.44 ± 0.02 ^bc^	51.48 ± 0.19 ^bc^	0.33 ± 0.003 ^b^
Diabetic FSP1	2.38 ± 0.02 ^bc^	49.65 ± 0.46 ^c^	0.27 ± 0.001 ^cd^
Diabetic FSP2	2.23 ± 0.02 ^c^	45.55 ± 1.0 ^d^	0.25 ± 0.005 ^c^
Diabetic FSP3	2.29 ± 0.06 ^c^	47.54 ± 0.33 ^e^	0.28 ± 0.004 ^de^

Negative control: nondiabetic and untreated rats, Positive control: diabetic rats, Diabetic FS1: diabetic rats received fenugreek seed powder at 5%, Diabetic FS2: diabetic rats received fenugreek seed powder at 7.5%, Diabetic FS3: diabetic rats received fenugreek seed powder at 10%, Diabetic FSP1: diabetic rats receiving 5% fenugreek sprouts, Diabetic FSP2: diabetic rats receiving 7.5% fenugreek sprouts, and Diabetic FSP3: diabetic rats receiving 10% fenugreek sprouts; HDL: high-density lipoprotein. Data are presented as mean ± SE (n = 6). Values with different letters in the same column significantly differ at *p* ≤ 0.05.

## Data Availability

The original contributions presented in the study are included in the article, further inquiries can be directed to the corresponding authors.
